# Differentiation under pressure: psychological pathways and latent profiles of GenAI dependence among graduate students

**DOI:** 10.3389/fpsyt.2026.1894754

**Published:** 2026-07-23

**Authors:** Xuejun Liu, Yi Zhang, Zijing Zhang, Renjie Li, Ágnes Bálint

**Affiliations:** 1 Institute of Education, Xiamen University, Xiamen, China; 2 Research Center for Grassroots Governance, School of Social Sciences, Tsinghua University, Beijing, China; 3 School of Economics and Management, Shandong Huayu University of Technology, Dezhou, China; 4 Doctoral School of Education, Faculty of Humanities and Social Sciences, University of Pécs, Pécs, Hungary; 5 Institute of Education, Faculty of Humanities and Social Sciences, University of Pécs, Pécs, Hungary

**Keywords:** critical thinking, GenAI dependence, research anxiety, research creativity, time pressure

## Abstract

**Purpose:**

As artificial intelligence becomes increasingly embedded in academic research, graduate students’ dependence on GenAI has emerged as a significant concern. This study aims to examine the psychological pathways linking time pressure with graduate students’ GenAI dependence, identify heterogeneous GenAI dependence profiles, and explore the demographic, cognitive, and creativity differences associated with these profiles.

**Methods:**

Drawing on survey data from 1,157 graduate students, this study employed PROCESS-based mediation analysis, latent profile analysis, multinomial logistic regression, and analysis of variance to examine the psychological pathways, user profiles, demographic correlates, and creativity differences associated with GenAI dependence.

**Results:**

(1) Time pressure was positively associated with GenAI dependence through two parallel indirect pathways: research anxiety (indirect effect = 0.162, accounting for 41.22% of the total effect) and performance expectancy (indirect effect = 0.108, accounting for 27.48%). The anxiety-related indirect pathway showed a larger association than the performance-expectancy pathway. (2) Four distinct types of GenAI dependence formation were identified: the All-low type (5.77%), the Balanced type (46.42%), the Dual-high/High-dependence type (39.90%), and the High-expectation/Low-anxiety type (7.91%). (3) Gender, academic year, and critical thinking were associated with profile membership. The High-expectation/Low-anxiety and Dual-high/High-dependence profiles showed similarly high creativity levels, both exceeding the Balanced and All-low profiles.

**Conclusion:**

The findings suggest that graduate students’ GenAI dependence is associated with both anxiety-related and performance-expectancy pathways under time pressure. The study identifies four distinct GenAI dependence profiles and highlights the role of critical thinking in differentiating these profiles. The High-expectation/Low-anxiety profile may represent a relatively adaptive form of GenAI engagement, although its creativity level was not significantly higher than that of the Dual-high/High-dependence profile.

## 1 Introduction

Generative AI, exemplified by ChatGPT, possesses capabilities in massive data processing, exceptional language understanding, and text-to-image generation. By enabling intelligent writing, optimizing academic searches, and assisting with routine tasks, it has emerged as an important research aid for graduate students (1). As artificial intelligence becomes increasingly embedded in academic research, the phenomenon of graduate students’ excessive dependence on GenAI has attracted growing scholarly concern. In this study, GenAI dependence is not treated as a clinical form of addiction, but as a continuum of reliance on generative AI tools in academic and research-related tasks. This continuum may include both functional reliance, such as using GenAI to improve efficiency and support research performance, and more problematic dependence, such as relying on GenAI to reduce anxiety, avoid cognitive effort, or substitute for independent academic judgment. Therefore, a higher level of GenAI dependence does not necessarily indicate maladaptive use in all cases; its meaning depends on the psychological conditions under which it emerges, such as research anxiety, performance expectancy, and critical thinking. Existing research suggests that GenAI use may have both enabling and risk-related implications. On the one hand, GenAI reflects the instrumental rationality of technological empowerment and efficiency-seeking. On the other hand, excessive or unreflective reliance on GenAI may be associated with academic inertia and may potentially weaken graduate students’ academic autonomy, innovative thinking, and problem-solving abilities during their formative academic years (2). Prior studies have also noted that the growing use of ChatGPT and other GenAI tools may be accompanied by dependence-related risks, including misuse and overreliance (3–5). Under high-pressure and competitive research environments, a sense of time urgency has become a prevalent psychological state among graduate students (6). When perceived time pressure coincides with the accessibility of GenAI tools, students may regard GenAI as a convenient means of accessing information, completing academic tasks, and responding to pressing research demands (7). In this context, time pressure may be positively associated with students’ reliance on GenAI in their academic work (8). However, this association may operate through different psychological pathways. Some students may experience research anxiety under time pressure and turn to GenAI for emotional relief, whereas others may view GenAI primarily as an efficiency-enhancing tool for achieving performance goals. The extent to which different types of GenAI-dependent users show higher levels of research creativity may depend on their higher-order cognitive capacities, such as critical thinking (9).

To capture individual differences, this study employed a mixed-analysis strategy, integrating PROCESS-based mediation analysis to examine the indirect associations linking time pressure, research anxiety, performance expectancy, and GenAI dependence, and latent profile analysis (LPA) to identify the GenAI dependence profiles of graduate students. Furthermore, it analyzed the demographic and cognitive correlates of these profiles and the differences in research creativity across different profiles. This study systematically addresses the following questions: First, how is time pressure associated with graduate students’ GenAI dependence through research anxiety and performance expectancy? Second, what GenAI dependence profiles can be identified among graduate students, and what characteristics define each profile? Third, which demographic and cognitive factors are associated with profile membership? Fourth, how does research creativity differ across different GenAI dependence profiles?

## 2 Literature review and research hypotheses

### 2.1 Time pressure and graduate students’ GenAI dependence

Time pressure refers to graduate students’ perception of time scarcity in completing academic and research-related tasks (10). Under the framework of Social Acceleration Theory, higher education has increasingly been characterized as “accelerated academia” (11). The economic logic of efficiency-seeking, the structural compression of academic time, and the cultural engine of time aversion collectively propel graduate students’ academic activities into a state of acceleration, thus turning to technological dependence to regain a sense of temporal efficiency (12). Within this context, graduate students may adopt different stress-coping strategies, including emotion-focused coping and problem-focused coping (13). When graduate students experience time pressure, on one hand, they develop research anxiety, defined as the negative emotions such as tension, unease, and irritability arising from perceived specific or potential threats related to unfinished research tasks, and may turn to on GenAI to alleviate the discomfort associated with anxiety (14). Other students may perceive GenAI primarily as an efficiency-enhancing tool and may use it to support academic efficiency, learning outcomes, and performance goals (8, 15).

Existing studies have provided preliminary evidence for this theoretical inference. Li and Jiang (10) demonstrated that time pressure significantly was positively associated with graduate students’ GenAI dependence. Similarly, Huang et al. (16) and Morales-García et al. (17) confirmed that anxiety positively and significantly was associated with AI dependence behavior (16, 17). Drawing on the I-PACE model, Zhang et al. (8) found that academic pressure was related to individuals’ AI dependence through the mediating role of performance expectancy (8). However, existing studies have primarily focused on undergraduate student populations and have often examined anxiety and expectancy within separate models. Drawing on stress-coping theory, this study explores how two distinct psychological pathways: emotion-focused coping and problem-focused coping, under time pressure may link with graduate students’ GenAI dependence. The following research hypotheses are proposed:

H1: Time pressure is positively associated with graduate students’ GenAI dependence.

H2: Research anxiety serves as an indirect pathway linking time pressure and graduate students’ GenAI dependence.

H3: Performance expectancy serves as an indirect pathway linking time pressure and graduate students’ GenAI dependence.

### 2.2 Heterogeneity in graduate students’ GenAI dependence profiles

Traditional research has largely relied on variable-centered approaches, which proceed from the assumption that samples are drawn from homogeneous populations and that all individuals follow similar psychological patterns. However, when facing similar levels of time pressure, different graduate students may adopt distinct motivational patterns, such as anxiety-driven or performance-driven pathway, which may correspond to different GenAI dependence behaviors. This individual heterogeneity can be more appropriately captured through person-centered approaches. In existing research, Chen et al. (18) identified four distinct thinking styles regarding artificial intelligence among college students: low engagement thinkers (19.6%), moderate engagement thinkers (23.4%), engaged and critical thinkers (23.9%), and high engagement thinkers (33.0%). The results revealed that negative academic emotions significantly predicted membership in all high engagement groups, with the strongest effect observed among high engagement thinkers. Test anxiety also emerged as a significant predictor for this group (18). Zhai (19) the distinct latent profiles of Chinese as a foreign language learners in Malaysia regarding their beliefs and engagement in AI-assisted Chinese learning. Using latent profile analysis, three profiles were identified: Enthusiastic but Limited Belief, Moderate Belief and Engagement, and Strong Belief and Engagement. Chi-square analyses revealed significant demographic differences across these groups in gender, AI learning experience, AI learning frequency, and trust levels in AI tools (19). Ishmuradova et al. (20) identified five distinct profiles of university students regarding their intentions to acquire AI knowledge: cautious participants, enthusiastic advocates, reserved skeptics, pragmatic acceptors, and detached critics. Among these, enthusiastic advocates exhibited the highest intention to acquire AI knowledge, while detached critics exhibited the lowest (20).

This study employs Latent Profile Analysis (LPA) to simultaneously incorporate four variables, time pressure, research anxiety, performance expectancy, and GenAI dependence into the analysis, aiming to identify distinct patterns of GenAI dependence profiles among graduate students. Accordingly, the following proposition is advanced:

H4: Graduate students’ GenAI dependence can be categorized into several latent profiles with distinct characteristics.

### 2.3 Critical thinking and GenAI dependence profiles

Critical thinking refers to the ability to analyze, evaluate, question, and creatively solve problems when acquiring and processing information (21). In the context of the increasing integration of GenAI into academic learning, critical thinking may help students maintain cognitive autonomy and engage with AI-generated content in a more reflective and evaluative manner. For example, Buçinca et al. (22) demonstrated that, in the context of AI-assisted decision-making, cognitive forcing strategies have been shown to encourage conscious and analytical thinking, effectively reducing overreliance (22). Hou et al. (23) confirmed that critical thinking encourages learners to adopt a more reflective, cautious, and collaborative approach to AI use, by strengthening the positive role of AI literacy while mitigating the blind conformity that may arise from overtrust (24).

In sum, existing research has predominantly treated critical thinking as a “consequence” rather than an “antecedent” of GenAI dependence. The present study posits that critical thinking may serve as a predictor of the types of GenAI dependence patterns that graduate students develop. Specifically, individuals with higher levels of critical thinking are more likely to critically evaluate the value and limitations of GenAI, employing it as a strategic tool for achieving performance goals rather than as an emotional outlet for alleviating anxiety. In contrast, those with lower levels of critical thinking may be more prone to uncritically accepting GenAI-generated output, rendering them more susceptible to anxiety-driven dependence patterns. Accordingly, the following hypothesis is proposed:

H5: Critical thinking is significantly associated with graduate students’ GenAI dependence profiles.

### 2.4 GenAI dependence profiles and research creativity

The question of whether GenAI dependence constitutes a detrimental cognitive shortcut or an empowering tool for research creativity has sparked considerable debate. One perspective argues that GenAI dependence undermines human autonomy and creativity (25). Another perspective contends that GenAI serves as a powerful tool capable of enhancing researchers’ efficiency and innovative capacity (26). Addressing this question, Yang et al. (27) found that AI dependence among university students significantly increases individual cognitive inertia, thereby diminishing their innovative capacity, with cognitive dependence exerting a stronger effect than instrumental dependence (27). Yao et al. (28) suggested that existential dependence on large language models negatively affects researchers’ creativity by triggering anxiety, whereas functional dependence positively affects researchers’ creativity by enhancing psychological availability (28). Ma et al. (29) suggested that the frequency of AIGC use significantly promotes graduate students’ research creativity and exhibits a significant nonlinear relationship with their research output. Furthermore, the effects of AIGC use and its frequency on graduate students’ research creativity demonstrate considerable heterogeneity across different groups (29).

These studies reveal that the relationship between GenAI dependence and graduate students’ research creativity is not simply linear, with the typologies of dependence formation and accompanying psychological states playing a significant role. That is, regarding the relationship between GenAI dependence and research creativity, what matters lies not in the level of dependence perse, but in the manner of dependence. Different GenAI dependence profiles exhibit significant differences in research creativity. Accordingly, it is hypothesized that:

H6: Research creativity differs significantly across graduate students’ GenAI dependence profiles.

**Figure 1:**
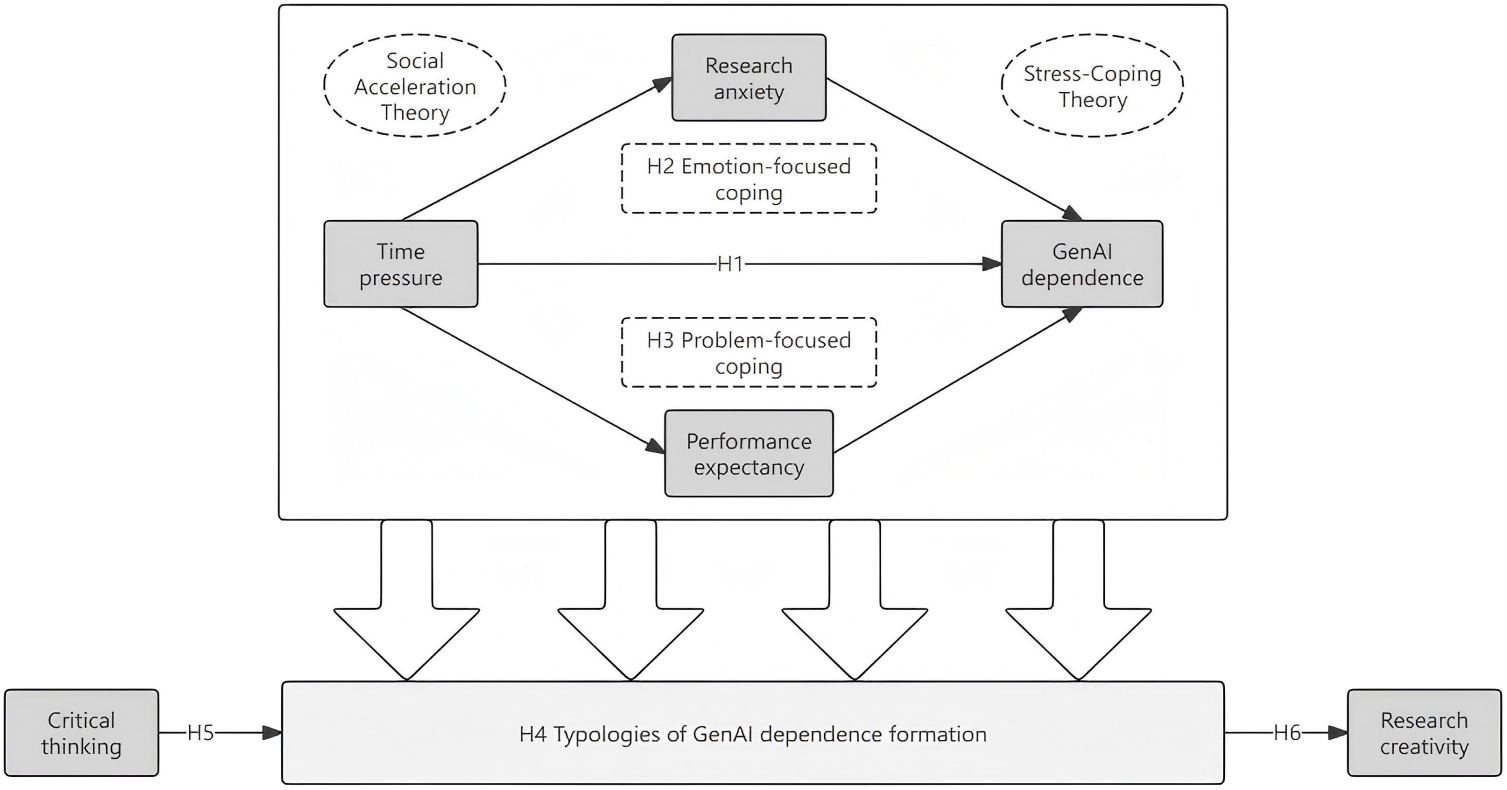
Mediation Mode.

The overall theoretical model of this study is shown in **Figure 1**.

## 3 Research design

### 3.1 Research participants

This study employed a random sampling method to administer an online survey to graduate students from universities in Shaanxi, Shandong, Fujian and other provinces. A total of 1,277 questionnaires were collected. After excluding invalid responses due to patterned answering, excessively short or long completion times, or failure to pass attention check questions, 1,157 valid questionnaires were obtained, yielding an effective response rate of 90.603%. This study was conducted in accordance with established ethical principles. As all participants were graduate students aged 18 years or older and capable of making informed judgments, they were provided with comprehensive information regarding the objectives, procedures, and data usage of this survey at the outset, and informed consent was obtained prior to their participation. Among the valid sample, there were 553 males (47.796%) and 604 females (52.204%). In terms of degree type, there were 401 academic master’s students (34.659%), 523 professional master’s students (45.203%), 175 academic doctoral students (15.125%), and 58 professional doctoral students (5.013%). Regarding academic year, 450 were first-year students (38.894%), 332 were second-year students (28.695%), 255 were third-year students (22.040%), 96 were fourth-year students (8.297%), and 24 were from other years (2.074%). By academic discipline, 121 students were from the humanities and arts (10.458%), 228 from social sciences and law (19.706%), 235 from natural sciences (20.311%), 261 from engineering (22.558%), 70 from agriculture (6.050%), 153 from medicine (13.224%), 50 from interdisciplinary and emerging fields (4.322%), and 39 from other majors (3.371%). This sample size surpasses the 5:1 parameter-to-respondent ratio recommended for SEM (30), ensuring statistical robustness.

### 3.2 Research instruments

The time pressure scale developed by Maruping et al. (31) was employed, comprising 3 items measured on a 5-point Likert scale ranging from 1 (“strongly disagree”) to 5 (“strongly agree”). Higher scores indicate greater perceived time pressure (31). In this study, the Cronbach’s α coefficient for this scale was 0.746. Given that the scale is unidimensional and consists of only three items, the model is just-identified (a saturated model), resulting in zero degrees of freedom (χ² = 0, df = 0). Consequently, conventional goodness-of-fit indices such as CFI and RMSEA could not be computed. In this context, following the recommendations of Hair et al. (32), we shifted our analytical focus to the component fit indices of the measurement model to examine the scale’s convergent validity 31. The analysis revealed that all items exhibited significant standardized factor loadings (p < 0.001), indicating that the observed variables effectively reflect their underlying latent construct.

Convergent validity was further assessed, yielding an average variance extracted (AVE) of 0.517 and a composite reliability (CR) of 0.756. According to the criteria recommended by Fornell and Larcker (33) and Hair et al. (32), an AVE greater than 0.5 indicates that the latent construct explains more than 50% of the variance in its indicators, while a CR greater than 0.7 signifies good internal consistency (32, 33). In this study, the AVE value slightly exceeded the threshold, while the CR value substantially surpassed the criterion, both of which collectively support the convergent validity of the scale. Therefore, although the scale could not yield global fit indices due to model identification constraints, its structural validity in this sample was confirmed through core indicators of convergent validity, rendering it suitable for subsequent hypothesis testing.

The research anxiety scale was adapted from Wang et al. (34), who developed the scale based on Rodell and Judge’s (2009) (35) conceptualization of anxiety and the Positive and Negative Affect Schedule developed by Watson and Clark (36). Research anxiety was defined as negative emotional experiences, such as worry, frustration, restlessness, and irritability, that arise when research tasks remain unfinished. The scale contains four items. The original Chinese items were used in the questionnaire, and their English translations are as follows: “When my research tasks remain unfinished, I always worry that I may not be able to complete them successfully”; “When my research tasks remain unfinished, I often complain about the strict assessment system”; “When my research tasks remain unfinished, I often feel frustrated”; and “When my research tasks remain unfinished, I often feel restless and irritable.” Responses were rated on a 5-point Likert scale ranging from 1 (“strongly disagree”) to 5 (“strongly agree”), with higher scores indicating higher levels of research anxiety. In this study, Cronbach’s α for the scale was 0.828. Confirmatory factor analysis indicated acceptable model fit: χ² = 22.035, df = 2, CFI = 0.99, TLI = 0.96, RMR = 0.03. Although this scale was adapted from prior Chinese research and showed acceptable reliability and CFA fit in the present sample, future studies should further validate it across broader graduate student populations and research contexts.

Drawing on the study by Acosta-Enriquez et al. (21), the performance expectancy scale developed by Zhang et al. (8) was adopted. The scale comprises five items measured on a 5-point Likert scale ranging from 1 (“strongly disagree”) to 5 (“strongly agree”), with higher scores indicating higher levels of performance expectancy. In this study, the Cronbach’s α coefficient for this scale was 0.885. Confirmatory factor analysis (CFA) results indicated a good model fit (χ² = 110.622, df = 5, CFI = 0.97, TLI = 0.93, RMR = 0.03).

Drawing on the AI dependence scale developed by Li et al. (37), this study employed an 18-item instrument comprising two dimensions: functional dependence and existential dependence. Items were rated on a 5-point Likert scale ranging from 1 (“strongly disagree”) to 5 (“strongly agree”), with higher scores indicating higher levels of AI dependence. In this study, the Cronbach’s α coefficient for this scale was 0.913. Confirmatory factor analysis (CFA) results indicated a good model fit (χ² = 676.92, df = 134, CFI = 0.95, TLI = 0.95, RMR = 0.05).

Drawing on the study by Acosta-Enriquez et al. (21), the critical thinking scale developed by Acosta-Enriquez et al. was adopted. The scale comprises five items measured on a 5-point Likert scale ranging from 1 (“strongly disagree”) to 5 (“strongly agree”), with higher scores indicating higher levels of critical thinking. In this study, the Cronbach’s α coefficient for this scale was 0.863. Confirmatory factor analysis (CFA) results indicated a good model fit (χ² = 71.12, df = 5, CFI = 0.97, TLI = 0.95, RMR = 0.03).

The research creativity scale for graduate students, adapted by Xie et al. (38) from a creativity scale, was employed. The scale comprises six items measured on a 5-point Likert scale ranging from 1 (“strongly disagree”) to 5 (“strongly agree”), with higher scores indicating higher levels of research creativity among graduate students. In this study, the Cronbach’s α coefficient for this scale was 0.885. Confirmatory factor analysis (CFA) results indicated a good model fit (χ² = 53.60, df = 9, CFI = 0.99, TLI = 0.98, RMR = 0.02).

### 3.3 Data analysis

SPSS 26.0 was used for reliability analysis, common method bias testing, descriptive statistics, correlation analysis, multinomial logistic regression, and analysis of variance (ANOVA). AMOS 26 was used to conduct confirmatory factor analysis (CFA) and measurement-model validation. The mediation model was tested using PROCESS Model 4 with 5,000 bootstrap samples (39). Time pressure was entered as the independent variable, GenAI dependence as the dependent variable, and research anxiety and performance expectancy as parallel mediators. Demographic variables were included as control variables. Mplus 8.3 was used for latent profile analysis. PROCESS-based mediation analysis and LPA served different analytical purposes. PROCESS was used to examine variable-centered indirect associations among time pressure, research anxiety, performance expectancy, and GenAI dependence, whereas LPA was used to identify person-centered configurations of these variables. Therefore, the LPA was not intended to replicate the mediation model, but to complement it by revealing heterogeneity among graduate students.

## 4 Research results

### 4.1 Common method bias test

Because all variables were collected from the same respondents using a self-reported questionnaire, common method bias was assessed using both Harman’s single-factor test and a confirmatory factor analysis (CFA) single-factor model. First, Harman’s single-factor test showed that five factors with eigenvalues greater than 1 were extracted without rotation, and the first factor accounted for 30.864% of the total variance, which was below the commonly used threshold of 40% (40). Second, to provide additional evidence beyond Harman’s test, a CFA single-factor model was estimated by loading all measurement items onto one latent factor. The results indicated a poor model fit: χ² = 15196.708, df = 702, χ²/df = 21.648, CFI = 0.446, TLI = 0.415, and RMR = 0.179. These fit indices were substantially below acceptable standards, suggesting that a single common factor could not adequately account for the covariance among the observed variables. In addition, although common method bias cannot be completely ruled out due to the cross-sectional self-report design, the results suggest that it is unlikely to be a serious threat to the main findings of this study.

### 4.2 Descriptive statistics and correlation analysis

Descriptive statistics, reliability, convergent validity, and correlation analysis results for the main variables are presented in **Table 1**. The CR values of all constructs ranged from 0.756 to 0.943, exceeding the recommended threshold of 0.70 and indicating acceptable composite reliability. The AVE values of most constructs were above 0.50. Although the AVE value of GenAI dependence was slightly below the conventional threshold, its CR value was high, suggesting that the construct still demonstrated acceptable composite reliability.

**Table 1:** Descriptive statistics and correlation analysis.

Variables	CR	AVE	M	SD	1	2	3	4	5	6
1. Time pressure	0.756	0.517	3.482	0.886	**0.719**					
2. Research anxiety	0.830	0.551	3.302	0.910	0.717**	**0.742**				
3. Performance expectancy	0.886	0.609	3.744	0.812	0.441**	0.332**	**0.780**			
4. GenAI dependence	0.943	0.483	3.352	0.690	0.507**	0.524**	0.472**	**0.695**		
5. Critical thinking	0.863	0.559	3.765	0.772	0.339**	0.230**	0.487**	0.305**	**0.748**	
6. Research creativity	0.886	0.751	3.635	0.748	0.319**	0.200**	0.444**	0.342**	0.825**	**0.867**

N = 1,157, **p < 0.01. The same applies below. Bold are the square roots of AVE.

In terms of correlations, time pressure was significantly and positively correlated with research anxiety (r = 0.717, p < 0.01), performance expectancy (r = 0.441, p < 0.01), GenAI dependence (r = 0.507, p < 0.01), critical thinking (r = 0.339, p < 0.01), and research creativity (r = 0.319, p < 0.01). Research anxiety was significantly and positively correlated with performance expectancy (r = 0.332, p < 0.01), GenAI dependence (r = 0.524, p < 0.01), critical thinking (r = 0.230, p < 0.01), and research creativity (r = 0.200, p < 0.01). Performance expectancy was also significantly and positively correlated with GenAI dependence (r = 0.472, p < 0.01), critical thinking (r = 0.487, p < 0.01), and research creativity (r = 0.444, p < 0.01). GenAI dependence was significantly and positively correlated with critical thinking (r = 0.305, p < 0.01) and research creativity (r = 0.342, p < 0.01). Critical thinking showed a strong positive correlation with research creativity (r = 0.825, p < 0.01).

Overall, the four variables used as latent profile indicators—time pressure, research anxiety, performance expectancy, and GenAI dependence—were significantly correlated with each other, providing a statistical basis for subsequent mediation analysis and latent profile analysis. At the same time, the relatively strong correlation between critical thinking and research creativity suggests that their empirical distinction should be interpreted with caution and further supported by additional discriminant validity evidence.

### 4.3 Mediation effect test

The four variables, time pressure, research anxiety, performance expectancy, and GenAI dependence, were all significantly positively correlated with each other, providing an empirical basis for further mediation analysis. Data analysis was conducted using Model 4 of the PROCESS macro in SPSS, employing bootstrap sampling (with 5,000 resamples). Time pressure was entered as the independent variable, GenAI dependence as the dependent variable, research anxiety and performance expectancy as mediator variables, and demographic variables as control variables.

**Figure 2:**
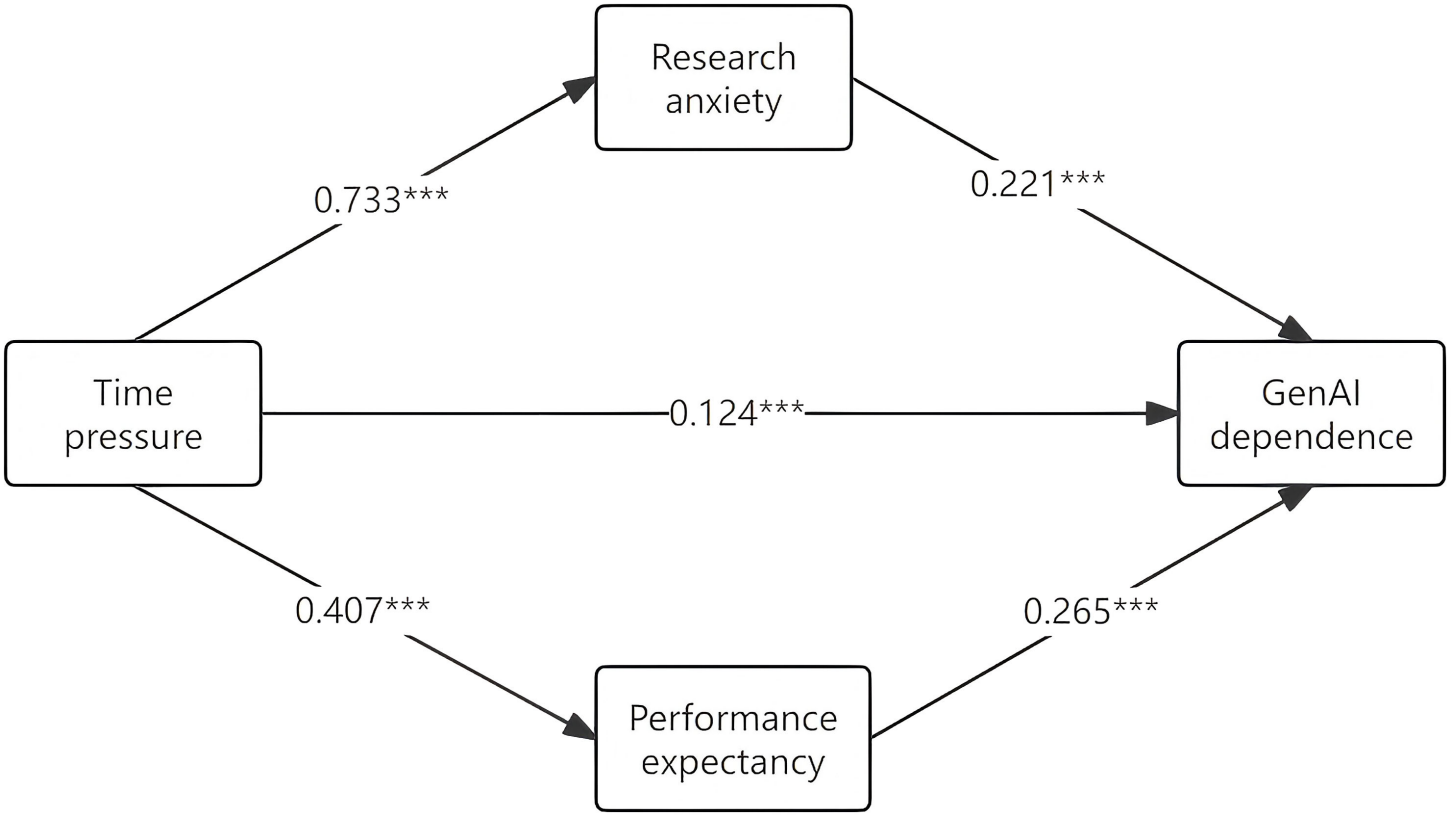
Theoretical model diagram.***p < 0.001.

The path coefficients are presented in **Figure 2**. Time pressure was significantly and positively associated with GenAI dependence (β = 0.124, p < 0.001), research anxiety (β = 0.733, p < 0.001), and performance expectancy (β = 0.407, p < 0.001). Research anxiety (β = 0.221, p < 0.001) and performance expectancy (β = 0.265, p < 0.001) were also significantly and positively associated with GenAI dependence.

The mediation effects are presented in **Table 2**. The direct association between time pressure and graduate students’ GenAI dependence was significant, with an effect size of 0.124, accounting for 31.552% of the total effect. Two significant indirect pathways linking time pressure with GenAI dependence were identified. The indirect pathway through research anxiety was significant, with an indirect effect of 0.162, accounting for 41.221% of the total effect. The indirect pathway through performance expectancy was also significant, with an indirect effect of 0.108, accounting for 27.481% of the total effect.

**Table 2:** Bootstrap test results of mediating effects.

Mode of effect	Path	Effect value	Effect proportion	SE	95% CI
Direct effect	Time pressure → GenAI dependence	0.124	31.552%	0.026	[0.072, 0.176]
Mediating effect	Time pressure → Research anxiety → GenAI dependence	0.162	41.221%	0.021	[0.121, 0.204]
	Time pressure → Performance expectancy → GenAI dependence	0.108	27.481%	0.014	[0.082, 0.136]
Total indirect effect		0.270	68.702%	0.025	[0.221, 0.318]
Total effect		0.393	100%	0.019	[0.356, 0.431]

Demographic variables were included as covariates in the PROCESS mediation model to reduce potential confounding. Because these covariates were not the theoretical focus of the mediation analysis, their coefficients are not discussed in detail in the main text; the main indirect effects remained significant after controlling for these variables.

### 4.4 Identification of GenAI dependence profiles among graduate students

Using the four variables, graduate students’ time pressure, research anxiety, performance expectancy, and GenAI dependence, as analysis indicators, latent profile analysis was conducted with Mplus 8.3, hypothesizing one to five classes for the formation types of graduate students’ GenAI dependence. The model fit indices are summarized in **Table 3**. The results indicated that as the number of classes increased, the AIC, BIC, and aBIC values decreased progressively. The Entropy values for the three-class, four-class, and five-class solutions were all above 0.8, and both the LMRT and BLRT were statistically significant for each class solution. Five-class and higher-order models were overly complex and lacked practically meaningful interpretation. Moreover, the five-class solution exhibited two classes with highly overlapping characteristics, posing challenges for effective differentiation. After comprehensive comparison, the four-class model demonstrated statistically sound fit indices, exhibited clearer class characteristics, and offered reasonable substantive interpretability. Therefore, this study ultimately determined the four-class solution as the optimal model.

**Table 3:** Summary of fitting index information for potential profile analysis.

LL	AIC	BIC	a BIC	Entropy	pLMR	pBLRT	Class proportion
-5645.662	11307.323	11347.752	11322.341				
-5134.924	10295.848	10361.545	10320.252	0.739	<0.001	<0.001	0.420, 0.580
-4871.223	9778.447	9869.411	9812.237	0.831	<0.001	<0.001	0.105, 0.480, 0.415
-4712.169	9470.338	9586.570	9513.515	0.855	<0.001	<0.001	0.058, 0.464, 0.399, 0.079
-4669.720	9395.440	9536.940	9448.003	0.877	<0.001	<0.001	0.056, 0.074, 0.445, 0.402, 0.022

To further evaluate the classification quality of the selected four-profile solution, average posterior probabilities were examined, as shown in **Table 3A**. The average posterior probabilities for the All-low profile, Balanced profile, Dual-high/High-dependence profile, and High-expectation/Low-anxiety profile were 0.958, 0.910, 0.923, and 0.909, respectively. All values exceeded the recommended threshold of 0.70, indicating acceptable classification accuracy and sufficient separation among the four profiles.

**Table 3a:** Classification quality of the selected four-profile solution.

Profile	Estimated n	Estimated proportion	Most-likely n	Average posterior probability
All-low profile	66.71	5.77%	66	0.958
Balanced profile	537.10	46.42%	541	0.910
Dual-high/High-dependence type	461.62	39.90%	463	0.923
High-expectation/Low-anxiety profile	91.57	7.91%	87	0.909

Estimated n and estimated proportion were based on the estimated posterior probabilities. Most-likely n was based on the most likely latent class membership. Average posterior probabilities are reported on the diagonal of the classification probability matrix.

**Figure 3:**
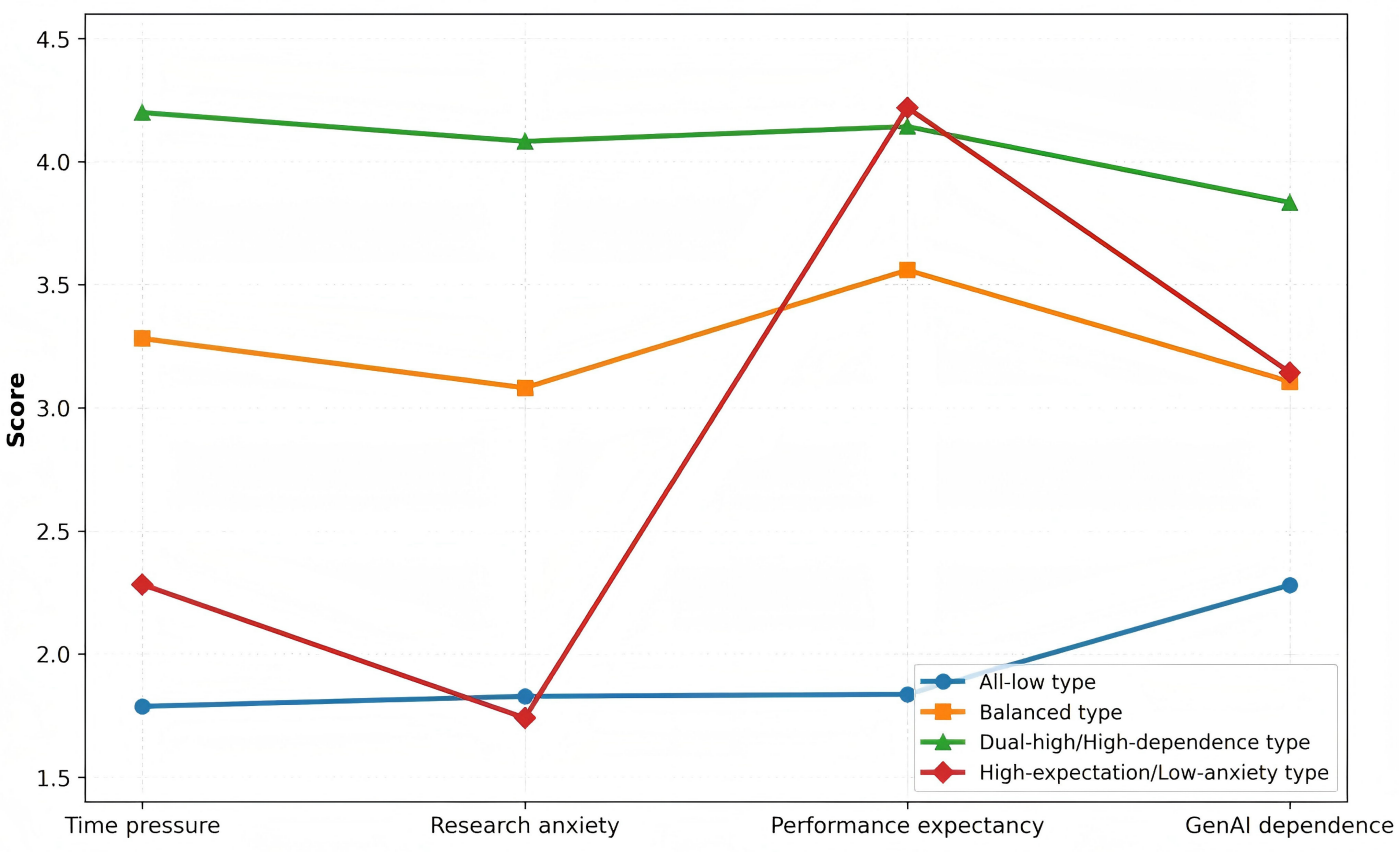
Potential profile characteristics of GenAI dependency formation types among different graduate students.

Using time pressure, research anxiety, performance expectancy, and GenAI dependence as profile indicators, latent profile analysis was conducted in Mplus 8.3 to estimate one- to five-profile solutions. Based on the scores of the four indicators, the four GenAI dependence profiles were named as follows. The profile characteristics are shown in **Figure 3**.

1. All-low type. This group of graduate students exhibited the lowest levels across all four variables, accounting for 5.77% of the sample. Their scores on time pressure, research anxiety, and performance expectancy were all below the midpoint of the scale, and their level of GenAI dependence was also within a low range. This represents a comprehensively low-involvement group, characterized by a weak perception of time pressure, a low level of research anxiety, a low expectation that using GenAI will enhance their academic efficiency, learning outcomes, and overall performance, and consequently, the lowest level of GenAI dependence.
2. Balanced type. This group represents the largest segment of the sample, comprising 46.42% of graduate students. Their scores on all four variables were slightly above the midpoint of the scale, exhibiting a balanced and moderate pattern. Overall, there were no substantial differences among the variables, and no pronounced extreme characteristics were observed. This group of graduate students experienced moderate levels of time pressure, exhibited a moderate degree of research anxiety, held moderate expectations that GenAI could enhance their research performance, and demonstrated a moderate level of GenAI dependence.
3. Dual-high/High-dependence type. This group represents the second largest segment of the sample, accounting for 39.90% of graduate students. They exhibited high levels across three variables—time pressure, research anxiety, and performance expectancy—and demonstrated the highest level of GenAI dependence among all four types. This represents a highly involved group, characterized by individuals experiencing significant time pressure alongside high levels of research anxiety, holding strong expectations that using GenAI will enhance their academic efficiency, learning outcomes, and overall performance, ultimately resulting in the most pronounced GenAI dependence. The label “Dual-high” refers specifically to the simultaneous elevation of the two psychological pathway variables—research anxiety and performance expectancy—rather than to the total number of elevated indicators. In this profile, time pressure was also high and functioned as the contextual stressor associated with these two psychological pathways.
4. High-expectation/Low-anxiety type. This group comprises 7.91% of the sample and exhibits the most distinctive contrast pattern, characterized by exceptionally high performance expectancy, the highest among the four types, coupled with remarkably low research anxiety, the lowest among the four types. Their time pressure is also relatively low, while their GenAI dependence is at a moderately high level. This group of graduate students holds strong expectations that using GenAI will enhance their academic efficiency, learning outcomes, and overall performance, while experiencing low levels of pressure and anxiety, and moderately utilizing GenAI tools to assist in their research.

### 4.5 Analysis of differences across typologies of graduate students’ GenAI dependence

#### 4.5.1 Demographic differences in GenAI dependence profiles among graduate students

The sample distribution of GenAI dependence profiles across demographic variables is presented in **Table 4**. Using the Balanced profile as the reference category, a multinomial logistic regression was conducted to examine the associations between demographic variables and GenAI dependence profiles. To address the sparse-cell problem in the previous model, first-year students were used as the reference category for academic year, and the small “Other” academic-year category was excluded from the regression analysis. In addition, the “Interdisciplinary and emerging fields” and “Other majors” categories were combined when analyzing academic discipline to reduce sparse-cell estimation problems.

**Table 4:** Sample distribution of GenAI dependence profiles by demographic variables.

Variables		All-low type	Balanced type	Dual-high/high-dependence type	High-expectation/low-anxiety type	Total
Gender	Male	39	230	233	51	553
	Female	27	311	230	36	604
Degree Type	Academic Master’s	13	223	130	35	401
	Professional Master’s	29	234	220	40	523
	Academic Doctoral	19	54	93	9	175
	Professional Doctoral	5	30	20	3	58
Academic Year	First-year	24	264	130	32	450
	Second-year	20	126	158	28	332
	Third-year	11	104	126	14	255
	Fourth-year	11	30	43	12	96
	Other	0	17	6	1	24
Discipline	Humanities & Arts	13	54	47	7	121
	Social Sciences & Law	8	95	110	15	228
	Natural Sciences	7	97	106	25	235
	Engineering	13	143	84	21	261
	Agriculture	10	26	27	7	70
	Medicine	12	82	52	7	153
	Interdisciplinary & Emerging Fields	3	19	24	4	50
	Other Majors	0	25	13	1	39

The revised results are shown in **Table 5**. Gender and academic year were significantly associated with profile membership. Compared with female students, male students were more likely to belong to the All-low profile rather than the Balanced profile (OR = 1.722, 95% CI [1.009, 2.939], p = 0.046), and were also more likely to belong to the High-expectation/Low-anxiety profile rather than the Balanced profile (OR = 1.746, 95% CI [1.088, 2.803], p = 0.021). In terms of degree type, academic master’s students were less likely than professional doctoral students to belong to the All-low profile rather than the Balanced profile (OR = 0.325, 95% CI [0.107, 0.991], p = 0.048). However, this result should be interpreted cautiously because the effect was marginal.

**Table 5:** Multinomial logistic regression results for demographic predictors of GenAI dependence profiles.

Variable	All-low profile vs. Balanced profile OR (95% CI)	p	Dual-high/high-dependence profile vs. Balanced profile OR (95% CI)	p	High-expectation/low-anxiety profile vs. Balanced profile OR (95% CI)	p
Gender						
Male vs. Female	1.722(1.009,2.939)	0.046	—	—	1.746 (1.088, 2.803)	0.021
Degree type						
Academic master’s vs. Professional doctoral	0.325(0.107,0.991)	0.048	—	—	—	—
Academic year						
Second-year vs. First-year	—	—	2.279 (1.646, 3.154)	<.001	—	—
Third-year vs. First-year	—	—	2.159 (1.529, 3.047)	<.001	—	—
Fourth-year vs. First-year	2.673(1.157,6.173)	0.021	2.404 (1.423, 4.059)	0.001	3.142 (1.432, 6.897)	0.004

The Balanced profile served as the reference category for the dependent variable. First-year students served as the reference category for academic year. The “Other” academic-year category was excluded from the multinomial logistic regression because of sparse cells. For academic discipline, “Interdisciplinary and emerging fields” and “Other majors” were combined to avoid sparse-cell estimation problems. Only statistically significant estimates are displayed. “—” indicates p ≥.05. OR, odds ratio; CI, confidence interval.

Academic year showed a clearer pattern. Compared with first-year students, second-year students (OR = 2.279, 95% CI [1.646, 3.154], p < 0.001), third-year students (OR = 2.159, 95% CI [1.529, 3.047], p < 0.001), and fourth-year students (OR = 2.404, 95% CI [1.423, 4.059], p = 0.001) were more likely to belong to the Dual-high/High-dependence profile rather than the Balanced profile. Fourth-year students were also more likely than first-year students to belong to the All-low profile (OR = 2.673, 95% CI [1.157, 6.173], p = 0.021) and the High-expectation/Low-anxiety profile (OR = 3.142, 95% CI [1.432, 6.897], p = 0.004). These findings suggest that academic year is an important demographic correlate of GenAI dependence profiles, especially for the Dual-high/High-dependence profile. Academic discipline did not show a significant association with profile membership after category consolidation.

#### 4.5.2 Critical thinking differences across GenAI dependence profiles

With the Balanced type as the reference group, multinomial logistic regression was conducted, and the results are presented in **Table 6**. The overall model was statistically significant (χ² = 243.669, df = 3, p < 0.001), with a Nagelkerke R² of 0.215, indicating that critical thinking was significantly associated with GenAI dependence profile membership. The results showed that the critical thinking scores across the four types ranked as follows: the High-expectation/Low-anxiety type scored the highest, followed by the Dual-high/High-dependence type, the Balanced type ranked in the middle, and the All-low type scored the lowest. Critical thinking levels varied across the different types. Compared to the Balanced type, critical thinking was significantly negatively associated with the All-low type (OR = 0.285, 95% CI [0.207, 0.394], p < 0.001), indicating that graduate students with higher levels of critical thinking are less likely to be classified into the All-low type. In contrast, higher critical thinking was associated with a greater likelihood of belonging to the High-expectation/Low-anxiety profile and the Dual-high/High-dependence profile rather than the Balanced profile.

**Table 6:** Multinomial logistic regression results for critical thinking and GenAI dependence profiles.

Comparison	OR	95% CI	p
All-low vs. Balanced	0.285	[0.207, 0.394]	<0.001
Dual-high/High-dependence vs. Balanced	2.890	[2.343, 3.565]	<0.001
High-expectation/Low-anxiety vs. Balanced	3.420	[2.320, 5.043]	<0.001

The Balanced profile served as the reference category. OR, odds ratio; CI, confidence interval.

This finding suggests that critical thinking does not simply correspond to lower GenAI dependence. Rather, as a higher-order cognitive capacity, critical thinking may be associated with more active, reflective, and strategic engagement with GenAI. Overall, critical thinking appears to be an important cognitive correlate that differentiates GenAI dependence profiles. In particular, higher critical thinking was linked not only to the High-expectation/Low-anxiety profile, but also to the Dual-high/High-dependence profile, suggesting that critical thinking can coexist with relatively high GenAI dependence under certain psychological configurations.

#### 4.5.3 Research creativity differences across GenAI dependence profiles

A one-way analysis of variance (ANOVA) was conducted with graduate students’ GenAI dependence profile as the grouping variable and research creativity as the dependent variable. The results are presented in **Table 7**. The ANOVA revealed significant differences in research creativity among the four types (F(3, 1153) = 93.284, p < 0.001), indicating that graduate students with different GenAI dependence profiles differed significantly in research creativity. *Post-hoc* comparisons revealed specific differences among the types. The High-expectation/Low-anxiety type exhibited the highest mean research creativity score (M = 3.92), which was significantly higher than that of the All-low type (MD = 1.333, p < 0.001) and the Balanced type (MD = 0.615, p < 0.001), but did not differ significantly from the Dual-high/High-dependence type. The Dual-high/High-dependence type also demonstrated a relatively high mean research creativity score (M = 3.77), significantly higher than the All-low type (MD = 1.190, p < 0.001) and the Balanced type (MD = 0.472, p < 0.001), with no significant difference compared to the High-expectation/Low-anxiety type. The Balanced type had a moderate mean research creativity score (M = 3.30), significantly higher than the All-low type (MD = 0.718, p < 0.001) and significantly lower than both the Dual-high/High-dependence type and the High-expectation/Low-anxiety type. The All-low type exhibited the lowest mean research creativity score (M = 2.58), significantly lower than all other types (all p < 0.001).

**Table 7:** Differences in research creativity across GenAI dependence profiles.

Variable	All-low type	Balanced type	Dual-high/high-dependence type	High-expectation/low-anxiety type	F	Post-hoc comparisons	p
Research creativity	2.58 ± 0.67	3.30 ± 0.67	3.77 ± 0.67	3.92 ± 0.67	93.284	4≈3>2>1	<0.001

The four types exhibited a clear gradient in research creativity, ordered as follows: High-expectation/Low-anxiety type ≈ Dual-high/High-dependence type > Balanced type > All-low type. The research creativity levels of the Dual-high/High-dependence type and the High-expectation/Low-anxiety type were similar, both significantly exceeding those of the Balanced type and the All-low type, while the Balanced type, in turn, demonstrated significantly higher research creativity than the All-low type. These results provide additional evidence for meaningful differences across the four-profile solution. However, because the High-expectation/Low-anxiety profile did not differ significantly from the Dual-high/High-dependence profile in research creativity, it should not be interpreted as a clearly superior profile. Rather, it may represent a relatively adaptive profile because it combines high performance expectancy and moderately high GenAI dependence with comparatively low research anxiety.

## 5 Conclusions and discussion

First, this study integrated Social Acceleration Theory and stress-coping theory to examine two psychological pathways—research anxiety and performance expectancy—that may link time pressure with graduate students’ GenAI dependence. It showed that time pressure was positively associated with graduate students’ GenAI dependence, and supported two significant indirect associations through research anxiety and performance expectancy. This finding offers a more nuanced theoretical perspective for understanding graduate students’ GenAI use under stressful conditions. Specifically, students reporting higher time pressure may differ in their coping-related psychological responses, including anxiety-related and performance-expectancy pathways. Those adopting emotion-focused coping may experience research anxiety and may turn to GenAI for emotional relief or task-related support. In contrast, those adopting problem-focused coping may perceive GenAI as a useful tool for supporting academic efficiency, learning outcomes, and performance goals. Notably, the indirect association of the emotion-focused coping pathway was larger than that of the problem-focused coping pathway. This suggests that, under time pressure, graduate students may be more strongly linked to GenAI dependence through anxiety-related responses than through performance-expectancy responses. This aligns with prior research, indicating that GenAI can provide both emotional and cognitive support (41). Anxiety has been identified as an important factor associated with individual GenAI dependence (42, 43), In academic contexts, performance expectancy has also been positively associated with researchers’ GenAI dependence (8).

Second, this study introduced a person-centered approach. Based on four variables, time pressure, research anxiety, performance expectancy, and GenAI dependence—latent profile analysis was employed to identify four heterogeneous types of graduate students’ GenAI dependence, namely, the All-low type, the Balanced type, the Dual-high/High-dependence type, and the High-expectation/Low-anxiety type. The sample sizes of the four types, ranked from highest to lowest, were as follows: the Balanced type (46.42%), the Dual-high/High-dependence type (39.90%), the High-expectation/Low-anxiety type (7.91%), and the All-low type (5.77%). The Balanced type exhibited no substantial differences among the variables, with no pronounced extreme characteristics. The Dual-high/High-dependence type demonstrated high levels across the three variables of time pressure, research anxiety, and performance expectancy, and exhibited the highest level of GenAI dependence among the four types. The High-expectation/Low-anxiety type showed relatively low time pressure, the highest performance expectancy among the four types, the lowest research anxiety, and a moderately high level of GenAI dependence. The All-low type exhibited low levels of time pressure, research anxiety, performance expectancy, and GenAI dependence. These findings extend variable-centered findings by showing that graduate students’ GenAI dependence is characterized by heterogeneous psychological configurations. In particular, the coexistence of relatively high time pressure, research anxiety, performance expectancy, and GenAI dependence appears to be an important pattern that deserves further scholarly and practical attention.

Third, this study further clarifies demographic, cognitive, and creativity differences across graduate students’ GenAI dependence profiles. In terms of demographic variables, the revised multinomial logistic regression results showed that gender and academic year were significantly associated with profile membership. Compared with female students, male students were more likely to be classified into the All-low profile and the High-expectation/Low-anxiety profile rather than the Balanced profile. Academic year showed a clearer pattern: compared with first-year students, second-, third-, and fourth-year students were more likely to belong to the Dual-high/High-dependence profile rather than the Balanced profile. This suggests that more advanced graduate students may be more likely to experience a configuration characterized by relatively high time pressure, research anxiety, performance expectancy, and GenAI dependence. In addition, fourth-year students were also more likely to belong to the All-low profile and the High-expectation/Low-anxiety profile, indicating that senior graduate students’ GenAI use may become more differentiated rather than uniformly more dependent. After category consolidation, academic discipline did not show a significant association with profile membership.

Critical thinking, as a crucial cognitive trait, was also associated with GenAI dependence profiles, but its role appears more nuanced than a simple protective factor against GenAI dependence. Students with higher levels of critical thinking were less likely to belong to the All-low profile and more likely to belong to the High-expectation/Low-anxiety and Dual-high/High-dependence profiles. This finding suggests that critical thinking does not necessarily reduce GenAI dependence in a linear manner. Instead, it may be associated with more active, reflective, and strategic engagement with GenAI. Under high time pressure and strong performance demands, students with stronger critical thinking may still rely heavily on GenAI, but this reliance may reflect purposeful academic engagement rather than passive or uncritical dependence. Therefore, the cultivation of critical thinking should not be seen as antithetical to GenAI use; rather, graduate students should be guided to use GenAI in a reflective, evaluative, and collaborative manner grounded in critical thinking (23).

Significant differences in research creativity were also observed across GenAI dependence profiles. Although the High-expectation/Low-anxiety profile showed the highest mean level of research creativity, its creativity score was not significantly different from that of the Dual-high/High-dependence profile. Therefore, this profile should be interpreted as a potentially adaptive and sustainable form of GenAI engagement rather than as a clearly superior profile. Drawing on Conservation of Resources theory, lower research anxiety may help preserve cognitive and psychological resources, while high performance expectancy may be associated with a stronger tendency to use GenAI strategically to support research tasks. Nevertheless, the relatively high creativity observed in the Dual-high/High-dependence profile also indicates that high GenAI dependence may not be uniformly detrimental, particularly when accompanied by strong academic engagement and performance-oriented use. These findings suggest that universities should not simply discourage GenAI use, but should promote responsible and reflective GenAI engagement by combining critical thinking training, anxiety management, and guidance on effective human-AI collaboration (44).

## 6 Practical implications

The findings of this study provide several practical implications for universities, supervisors, and graduate training programs. First, universities should avoid treating GenAI dependence as a uniformly negative phenomenon. The results suggest that GenAI dependence may appear in different psychological configurations. Therefore, rather than simply discouraging or prohibiting GenAI use, universities should develop clear guidelines for responsible, transparent, and ethical GenAI engagement in academic research. Such guidelines should clarify acceptable and unacceptable uses of GenAI in literature searching, academic writing, data analysis, idea generation, and research communication.

Second, graduate training programs should pay closer attention to the role of time pressure and research anxiety. The Dual-high/High-dependence profile indicates that some graduate students may experience high time pressure, high research anxiety, high performance expectancy, and relatively strong GenAI dependence simultaneously. For these students, GenAI use may be closely associated with emotional relief and pressure management. Universities and supervisors should therefore provide timely academic support, reasonable workload expectations, and psychological support services to help students manage research anxiety rather than relying excessively on GenAI as a coping tool.

Third, the role of critical thinking should be emphasized in GenAI-related training. The findings indicate that higher critical thinking is associated not only with the High-expectation/Low-anxiety profile but also with the Dual-high/High-dependence profile. This suggests that critical thinking does not necessarily reduce GenAI use or dependence in a simple linear manner. Instead, critical thinking may help students engage with GenAI more reflectively and strategically. Graduate programs should therefore incorporate training on evaluating AI-generated content, identifying hallucinations and biases, maintaining academic judgment, and integrating GenAI outputs with independent reasoning.

Fourth, universities may benefit from profile-based guidance rather than one-size-fits-all interventions. Students in the All-low profile may need support in developing basic GenAI literacy and understanding how AI tools can support research tasks. Students in the Balanced profile may benefit from general guidance on responsible and efficient GenAI use. Students in the Dual-high/High-dependence profile may require combined support in anxiety management, time management, and reflective AI use. Students in the High-expectation/Low-anxiety profile may serve as a useful reference for relatively adaptive GenAI engagement, as they appear to combine strong performance expectations with lower anxiety and moderately high GenAI dependence. Overall, the goal of graduate education should not be to eliminate GenAI use, but to promote responsible, reflective, and human-centered human-AI collaboration.

## 7 Limitations and future research

This study has several limitations that should be acknowledged. First, this study used a cross-sectional survey design. Although PROCESS-based mediation analysis was used to examine the indirect associations among time pressure, research anxiety, performance expectancy, and GenAI dependence, the temporal ordering among these variables cannot be established. Therefore, the findings should be interpreted as associations rather than causal effects. Future research could adopt longitudinal, experimental, or cross-lagged designs to examine how graduate students’ time pressure, psychological responses, and GenAI dependence evolve over time.

Second, all variables were measured using self-reported questionnaires collected from the same respondents at the same time point. Although Harman’s single-factor test and the CFA single-factor model suggested that common method bias was unlikely to fully account for the findings, this risk cannot be completely ruled out. Future studies could combine self-reported data with behavioral data, supervisor evaluations, GenAI usage logs, or objective indicators of research performance and creativity.

Third, although the research anxiety scale was adapted from prior research and showed acceptable reliability and CFA fit in the present sample, further validation is still needed. Future studies should continue to examine the scale’s content validity, measurement invariance, and applicability across different graduate student populations, disciplines, and academic cultures.

Fourth, the sample was drawn from graduate students in several Chinese provinces. Therefore, the generalizability of the findings to broader national or cross-cultural contexts should be interpreted with caution. Future research should include more diverse samples from different regions, institutional types, disciplines, and cultural backgrounds.

Fifth, although this study identified four GenAI dependence profiles, the interpretation of these profiles should remain cautious. In particular, the All-low profile and the High-expectation/Low-anxiety profile accounted for relatively small proportions of the sample. In addition, the creativity score of the High-expectation/Low-anxiety profile was not significantly different from that of the Dual-high/High-dependence profile. Future research should further test the stability, replicability, and developmental trajectories of these profiles in larger and more diverse samples.

Finally, this study examined critical thinking and research creativity using self-reported scales. The relatively strong correlation between these two constructs suggests that future studies should use multiple measurement approaches, such as performance-based critical thinking tasks, expert-rated research outputs, or longitudinal creativity indicators. Such approaches would provide more robust evidence regarding how GenAI use is associated with graduate students’ research creativity.

## Data Availability

The raw data supporting the conclusions of this article will be made available by the authors, without undue reservation.
